# Drugs prescribed by general practitioners according to age, gender and socioeconomic status after adjustment for multimorbidity level

**DOI:** 10.1186/s12875-014-0183-8

**Published:** 2014-11-25

**Authors:** Jessica Skoog, Patrik Midlöv, Anders Beckman, Jan Sundquist, Anders Halling

**Affiliations:** Department of Clinical Sciences in Malmö, Center for Primary Health Care Research, Lund University, SE-205 02 Malmö, Sweden; Stanford Prevention Research Center, Stanford University School of Medicine, Stanford, CA USA; Research Unit of General Practice, Institute of Public Health, University of Southern Denmark, J.B. Winsløws Vej 9A, DK-5000 Odense C, Denmark

**Keywords:** Prescription drug, Pharmacological treatment, Primary health care, General practitioner, Multimorbidity, Case-mix, Gender, Age, Income, Education, Socioeconomic status

## Abstract

**Background:**

Age, gender and socioeconomic status have been shown to be associated with the use of prescription drugs, even after adjustment for multimorbidity. General practitioners have a holistic and patient-centred perspective and our hypothesis is that this may reflect on the prescription of drugs. In Sweden the patient may seek secondary care without a letter of referral and the liability of the prescription of drugs accompanies the patient, which makes it suitable for this type of research. In this study we examine the odds of having prescription drug use in the population and the rates of prescription drugs among patients, issued in primary health care, according to age, gender and socioeconomic status after adjustment for multimorbidity level.

**Method:**

Data were collected on all individuals above 20 years of age in Östergötland county with about 400 000 inhabitants in year 2006. The John Hopkins ACG Case-mix was used as a proxy for multimorbidity level. Odds ratio (OR) of having prescription drugs issued in primary health care in the population and rates of prescription drug use among patients in primary health care, stated as incidence rate ratio (IRR), according to age, gender and socioeconomic status were calculated and adjusted for multimorbidity.

**Results:**

After adjustment for multimorbidity, individuals 80 years or older had higher odds ratio (OR 3.37 (CI 95% 3.22-3.52)) and incidence rate ratio (IRR 6.24 (CI 95% 5.79-6.72)) for prescription drug use. Male individuals had a lower odds ratio of having prescription drugs (OR 0.66 (CI 95% 0.64-0.69)), but among patients males had a slightly higher incidence rate of drug use (IRR 1.06 (CI 95% 1.04-1.09)). Individuals with the highest income had the lowest odds ratio of having prescription drugs and individuals with the second lowest income had the highest odds ratio of having prescription drugs (OR 1.10 (CI 95% 1.07-1.13)). Individuals with the highest education had the lowest odds ratio of having prescription drugs (OR 0.61 (CI 95% 0.54-0.67)).

**Conclusion:**

Age, gender and socioeconomic status are associated with large differences in the use of prescribed drugs in primary health care, even after adjustment for multimorbidity level.

**Electronic supplementary material:**

The online version of this article (doi:10.1186/s12875-014-0183-8) contains supplementary material, which is available to authorized users.

## Background

Previous studies have shown that drugs are prescribed unequally in the general population in Sweden, Europe, Canada and in the United States [1-4]. Individuals with low socioeconomic status use more prescription drugs compared to individuals with high socioeconomic status [5]. Males tend to use prescription drugs to a lower degree compared to females [6]. High age tends to increase the use of prescription drugs [7], even after adjustment for multimorbidity level [8]. In the above comparisons the prescribing of drugs in the total population has been evaluated. Few studies have been performed that only take into account the prescriptions issued by physicians working in primary health care, i.e. general practitioners (GPs).

Primary health care is performed in different socioeconomic environments. General practitioners handle patients with a holistic and patient-centred perspective and have a unique insight into patients’ context and are accustomed to consider differences in health [9]. Our hypothesis is that this approach may have an influence on the prescription of drugs. As in most Western European countries, the first-line of health care in Sweden is primary health care. In contrast to other Western Europeans countries in Sweden the GPs do not have a gatekeeping function. In case of health problems the individual cannot handle him- or herself, he or she is supposed to get in contact with his/her GP to get a primary evaluation of the health status. In case the GP needs a second opinion or if there is a need of larger resources to investigate the health issue or disease, the GP refers to a specialist. Nevertheless, the individual is allowed to seek secondary care, both at the emergency room and at specialist clinics, without a letter of referral. Only half of the patients who visit Swedish health care meet a GP [10]. When the patient seeks secondary care the liability regarding the prescription of drugs accompanies the patient, regardless if the patient was referred or not. Thus the prescription of drugs in Sweden, in contrast to other Western European countries, is divided between primary and secondary care, which makes Sweden quite suitable for this type of research.

This study further enables us to compare the drug prescriptions issued by all the physicians with the drug prescriptions issued only by GPs. With respect to the cost of prescription drugs this estimation is furthermore of high interest. The use of prescription drugs constitutes about 13% of the total cost of health care in Sweden, and in most parts of Sweden the cost liability for the drugs prescribed by the GPs is put on the individual primary health care centres [11]. To be able to affect the prescribing of drugs it is fundamental to know how drugs are prescribed in primary health care, and it is important to know about differences in drug prescribing between various groups.

The aim of this study was to investigate the odds of having prescription drugs among individuals in the population treated by GPs, and the rate of prescription drug use among patients treated by GPs depending on age, gender and socioeconomic status after adjusting for multimorbidity level.

## Methods

### Study population

Data were collected in 2006 from the total population aged 20 years or older in Östergötland county with about 400 000 residents. Östergötland county is situated 200 km southwest of Stockholm, and the age demography match corresponds to that of the rest of Sweden [12]. Data on the population’s age, gender and diagnoses in both primary and secondary care were obtained through the Care Data Warehouse in Östergötland (CDWÖ). The data in this register hold information on both public and private care. Information on this register has been described earlier [13]. The study was approved by the Research Ethics Committee at Linköping University (Dnr 147/05 and 29/06).

### Independent variables

Multimorbidity level was calculated using the Johns Hopkins Adjusted Clinical Groups (ACG) Case-Mix System, a system based on the theory that multimorbidity level corresponds to a certain need for healthcare resources. This system is based on the patients’ diagnoses, from both primary and secondary care, recorded during a defined period of time. Etiology, duration, method of diagnosis, treatment and need of specialised care are considered for each of the patients’ diagnoses. The ACG Case-Mix System has previously been described [14-17]. Individuals without need of health care according to the ACG Case-Mix system are placed in Resource Utilization Band 0 (RUB 0), and individuals with a very high degree of need for healthcare resources are placed in RUB 5.

We used income and education as our socioeconomic variables. The individual disposable income was divided into quartiles, from the lowest to the highest with equal number of individuals in each quartile. The individual income includes earnings from employment and business, and income transfers (e.g., pension payments, unemployment benefits, or paid sick leave) but not capital returns. The education variable was divided into four levels: 1. Primary school not completed (<9 years), 2. Primary school completed (9–10 years), 3. Secondary school (10–12 years), and 4. Higher education (>12 years). Information on individual level of income and education was obtained from Statistics Sweden. Information on educational level was to a high degree missing in people 70 years or older, therefore this group was excluded when the effect of educational level on prescription drug use was analysed.

### Dependent variable

The utilisation of prescription drugs, stated as Defined Daily Doses (DDDs), in 2006 issued by a GP was the dependent variable. Information concerning the use of prescription drugs on the individual level was acquired from the Swedish Prescribed Drug Register at the National Board of Health and Welfare [18]. This register collects the information from the National Corporation of Swedish Pharmacies (Apoteket AB). At the time of the study Apoteket AB had a monopoly on sales of prescription drugs, and all prescription drugs were tracked through Apoteket AB. DDD is defined by WHO as the assumed average maintenance dose per day for a drug used for its main indication in adults. It is a fixed unit of measurement that enables comparative research on prescription drugs [19]. Over the counter drugs were not included in this study.

### Statistics

We used STATA version 12 (Stata Corporation, Texas, USA) for statistical analyses. The best statistical model to define the data was considered to be zero-inflated negative binomial regression, since this model takes into account that a high number in the population do not use prescription drugs [20]. This model performs two analyses in parallel. One analysis is similar to logistic regression and answers the question on what the odds are for the individual to belong to the population with prescription drugs issued by GPs. This analysis gives odds ratios (OR). The other analysis is similar to Poisson regression and answers the question of what the effect is of increasing the independent variable, i.e. DDD, with one unit for the individuals who already have at least one DDD. This analysis gives an incident rate ratio (IRR). We generated two different models; Model 1 was adjusted for gender, age, multimorbidity (RUB) and income, and Model 2 was adjusted for gender, age, multimorbidity (RUB) and education.

In order to examine if the differences in the prescription of drugs were dependent on the primary healthcare centres (PHC), we performed a multi-level analysis. The analysis showed that only about 2% of the differences seen between various groups was dependent on the PHC level.

Due to possible interaction between the variables education and income we have analysed the data for each educational level, comprising individuals up to 70 years old. We have also analysed the data for each age level due to possible interaction between age and multimorbidity level.

In the following sections, when we refer to odds ratios we mean individuals in the population, i.e. people both with and without prescription drugs, and when we refer to incidence rate ratios we mean patients, i.e. people that already have at least one prescription drug.

## Results

The study comprised 313 977 individuals with an even sex distribution. A total of 46% had at least one prescription drug issued by a GP. About 231 million DDDs were collected from the pharmacies. Further characteristics of the study population are described in Table 1.

**Table 1:** **Characteristics of the study population’s drug use**

	**Collected prescription drugs in the total population**		**Collected prescription drugs in the total population issued by general practitioner**	
	**Yes**	**No**	**Yes**	**No**
**Variables**	**N (%)**	**N (%)**	**N (%)**	**N (%)**
All	205827 (66)	108150 (34)	145126 (46)	168851 (54)
Gender				
Female	121682 (77)	37021 (23)	85102 (54)	73601 (46)
Male	84145 (54)	71129 (46)	60024 (39)	95250 (61)
Age				
20-39	51582 (52)	47857 (48)	25953 (74)	73486 (26)
40-59	65365 (60)	43338 (40)	44584 (41)	64119 (59)
60-79	64388 (81)	15414 (19)	52494 (34)	27308 (66)
80-	24492 (94)	1541 (6)	22095 (85)	3938 (15)
Multimorbidity level				
0	26822 (26)	75013 (74)	16657 (16)	85178 (84)
1	30364 (69)	13491 (31)	17594 (40)	26261 (60)
2	51674 (80)	12913 (20)	35401 (55)	29186 (45)
3	82988 (93)	6595 (7)	63432 (71)	26151 (29)
4	10775 (99)	126 (1)	9196 (84)	1705 (16)
5	3204 (99.6)	12 (0.4)	2846 (88)	370 (12)
Income level				
1 (low)	55260 (70)	23185 (30)	40986 (52)	37459 (48)
2	59030 (75)	19415 (25)	43876 (56)	34569 (44)
3	48816 (62)	29630 (38)	32426 (41)	46020 (59)
4 (high)	42720 (55)	35724 (45)	27837 (35)	50607 (65)
Educational level*				
1 (low)	15377 (73)	5732 (27)	12166 (58)	8943 (42)
2	16292 (62)	10003 (38)	11070 (42)	15225 (58)
3	74886 (60)	50695 (40)	48366 (39)	77215 (61)
4 (high)	45296 (56)	35001 (44)	26552 (33)	53745 (67)

*N* – Number of observations.

*Including individuals up to 70 years old.

### Age

After adjustment for multimorbidity level, gender and income, age increased the odds ratios of having prescriptions drugs issued by GPs in the population, and it also increased the rate of prescription drug use among patients (Tables 2 and 3).

**Table 2:** **Odds ratios of having prescription drugs in the population issued in primary health care**

**Variables**	**OR (CI 95%)**	**p-value**	OR (CI 95%)*	**p-value**
Gender				
Females	1		1	
Males	0.66 (0.64-0.69)	<0.001	0.63 (0.60-0.66)	<0.001
Age				
20-39	1		1	
40-59	1.76 (1.72-1.79)	<0.001	1.71 (1.68-1.75)	<0.001
60-79	2.56 (2.49-2.62)	<0.001	2.33 (2.27-2.39)	<0.001
80-	3.37 (3.22-3.52)	<0.001		
Multimorbdity level				
RUB 0 (low)	1		1	
RUB 1	2.38 (2.32-2.44)	<0.001	2.48 (2.42-2.54)	<0.001
RUB 2	2.86 (2.79-2.93)	<0.001	2.95 (2.88-3.02)	<0.001
RUB 3	3.30 (3.21-3.39)	<0.001	3.39 (3.30-3.48)	<0.001
RUB 4	3.75 (3.63-3.87)	<0.001	3.86 (3.75-3.97)	<0.001
RUB 5 (high)	3.88 (3.73-4.04)	<0.001	4.01 (3.79-4.22)	<0.001
Income				
1 (low)	1		-	
2	1.10 (1.07-1.13)	<0.001	-	
3	0.98 (0.95-1.02)	0.375	-	
4 (high)	0.86 (0.81-0.91)	<0.001	-	
Education				
1 (low)	-		1	
2	-		0.85 (0.81-0.89)	<0.001
3	-		0.77 (0.73-0.81)	<0.001
4 (high)	-		0.61 (0.54-0.67)	<0.001

*Including individuals up to 70 years old.

**Table 3:** **Incidence rate ratios of prescription drug use among patients in primary health care**

**Variables**	**IRR (CI 95%)**	**p-value**	IRR (CI 95%)*	**p-value**
Gender				
Females	1		1	
Males	1.06 (1.04-1.09)	<0.001	0.99 (0.96-1.02)	0.674
Age				
20-39	1		1	
40-59	2.41 (2.31-2.52)	<0.001	2.29 (2.19-2.39)	<0.001
60-79	4.34 (4.12-4.57)	<0.001	3.78 (3.62-3.96)	<0.001
80-	6.24 (5.79-6.72)	<0.001		
Multimorbidity level				
RUB 0 (low)	1		1	
RUB 1	0.74 (0.71-0.78)	<0.001	0.69 (0.66-0.73)	<0.001
RUB 2	0.94 (0.90-0.98)	0.004	0.92 (0.88-0.97)	0.001
RUB 3	1.33 (1.29-1.37)	<0.001	1.35 (1.30-1.41)	<0.001
RUB 4	1.66 (1.58-1.74)	<0.001	1.89 (1.74-2.05)	<0.001
RUB 5 (high)	1.75 (1.63-1.86)	<0.001	2.26 (2.00-2.55)	<0.001
Income				
1 (low)	1		-	
2	1.00 (0.98-1.03)	0.811	-	
3	0.78 (0.75-0.81)	<0.001	-	
4 (high)	0.70 (0.68-0.72)	<0.001	-	
Education				
1 (low)	-		1	
2	-		0.97 (0.91-1.02)	0.227
3	-		0.82 (0.80-0.85)	<0.001
4 (high)	-		0.70 (0.67-0.73)	<0.001

*Including patients up to 70 years old.

### Gender

Males had lower odds ratio of having prescription drugs issued by GPs compared to females after adjustment for multimorbidity level, age and income (OR 0.66 (95% CI 0.64-0.69)) or education (OR 0.63 (95% CI 0.60-0.66)) (Table 2). The rates of prescription drug use were higher for male patients after adjustment for multimorbidity level, age and income (IRR 1.06 (95% CI 1.04-1.09)). The rates of prescription drug use were the same between the genders after adjustment for multimorbidity, age and education (IRR 0.99 (95% CI 0.96-1.02)) (Table 3).

### Income

Individuals with the highest level of income had the lowest odds ratio of having prescription drugs issued by GPs after adjustment for multimorbidity level, gender and age (OR 0.86 (95% CI 0.81-0.91)). Individuals with the second lowest level of income had the highest odds ratio of having prescription drugs (OR 1.10 (95% CI 1.07-1.13) and the individuals with the lowest level of income had the second highest odds ratio of having prescription drugs issued by GPs (Table 2). The result was nearly the same for the rates of prescription drug use among patients with the exception of that the patients with the lowest and the second lowest income level together had the lowest rates of prescription drug use (Table 3).

### Education

Individuals with the lowest level of education had the highest odds ratio of having prescription drugs issued by GPs after adjustment for multimorbidity level, gender and age. The odds ratios decreased with increasing levels of education with the lowest odds ratio of having prescription drugs at the highest level of education (OR 0.61 (95% CI 0.54-0.67). The associations were the same for the rates of prescription drug use among patients (Tables 2 and 3).

### Interactions

Individuals with the lowest level of income had the lowest odds ratio of having prescription drugs if they belonged to educational level 2 or above. The rates of prescription drug use in every income level followed the same pattern as in Model 1 when the data was analysed for each educational level (Additional file 1: Table S1).

The odds ratio of having prescription drugs and the rate of prescription drug use showed nearly the same pattern in every multimorbidity level in Model 1 when the data was analysed for each age level besides that both individuals and patients aged 20–39 in the highest multimorbidity level had higher odds ratios of having prescription drugs (OR 4.32 (95% CI 4.07-5.54) vs. OR 3.88 (95% CI 3.73-4.04)) and higher rates of prescription drug use (IRR 3.99 (95% CI 1.88-8.45) vs. IRR 1.75 (95% CI 1.63-1.86)) (Additional file 1: Table S2).

## Discussion

In this study we have examined the use of prescription drugs issued in primary health care according to age, gender, income or education after adjustment for multimorbidity level. Our main findings were that age increased the odds ratio of having prescription drugs, despite adjustment for multimorbidity level. We found that males had lower odds ratios of having prescription drugs compared to females. The differences in the socioeconomic groups were also substantial, where people with the highest income level had the lowest prescription drug use and people with the second to lowest income level had the highest prescription drug use. People with the lowest educational level had the highest prescription drug use.

### Age

The finding that age increased both the odds ratio of having prescription drugs and the rate of prescription drug use has previously been shown, when the total prescribing of drugs has been examined [3]. In this study we have only examined the prescription drugs issued in primary health care, and despite the fact that we have adjusted for multimorbidity level, higher age seems to lead to higher use of prescription drugs.

GPs may have different approaches to treatment of patients with a new diagnosis depending on age. It is probably more likely that younger people with a new diagnosis of for example hypertension are recommended changes in lifestyle, while older people may be more likely to be recommended a prescription drug. This may partly explain higher odds ratios of prescription drug use among the elderly. Some diagnoses are progressive, for instance diabetes mellitus, where worsening is expected with higher age [21]. Under these circumstances increasing prescription drug use among elderly is expected and may partly explain the higher rate of prescription drug use among the patients. The prescribing cascade may help to explain, why the elderly use more prescription drugs despite adjustment for multimorbidity level. The prescribing cascade is described to start as an adverse drug reaction that is misinterpreted as a new diagnosis. A new drug is prescribed to treat this “new” diagnosis, and at worst an adverse drug reaction against the new prescription drug is once again misinterpreted as a new diagnosis and another drug is prescribed [22]. With higher age and more diagnoses it is more likely that the elderly are put at risk of this prescribing cascade. The above may to some extent explain why the elderly have higher odds ratios and rate of prescription drug use, which calls for better quality in drug treatment in the elderly.

The difference in odds ratio of having prescription drugs according to age is less prominent, when prescription drugs issued in primary health care are compared with prescription drugs in the total population [8]. Since many elderly with chronic diseases are treated in primary health care in Sweden, we would rather expect that the differences would be greater in primary health care compared to studies where the total population was examined. It could be that GPs have a more holistic approach and that GPs are better at evaluating prescription drugs [23]. It could also be that GPs to a lower extent tend to follow treatment guidelines [24].

### Gender

Males had significantly lower odds ratio of having prescription drugs compared to females, despite adjustment for multimorbidity level. This situation has been shown before in studies carried out on the total population [6,25], but rarely with adjustment for multimorbidity level and rarely only in a primary healthcare population.

Females tend to utilise health care more often than males [26], which may partly explain the gender difference. Females also tend to seek more preventive care than males [27], which may further contribute to the gender difference. There is a gender difference in which diagnoses females and males are diagnosed with, and a former study indicates that this gender difference in morbidity may partly explain the gender difference in odds of having prescription drugs [28]. Former studies have shown that there is variability between how physicians prescribe drugs and it is possible that this may affect the gender difference [29]. It is puzzling that the odds ratio for males in the population to have at least one prescription drug is quite low (OR 0.66), while males among patients in parallel have a higher rate of prescription drug use (IRR 1.06). This could be interpreted as the barrier to initiate prescription drug treatment being higher for males. It is also possible that there is a gender difference in compliance to drug treatment, which has been indicated in some studies [30,31].

The gender difference regarding odds ratios of having prescription drugs is less distinct when prescription drug use issued in the primary health care is compared with the total prescription drug use [8]. This could partly be explained by the fact that in Sweden, at the time of the study, oral contraceptive drugs were not prescribed by GPs in primary health care, but mainly issued by midwives belonging to secondary care.

### Income

Individuals and patients with the highest level of income had the lowest odds ratio and rate of prescription drug use issued in primary health care. Individuals with the second to lowest income had the highest odds ratio of having prescription drugs.

Despite adjustment for multimorbidity level there are substantial differences between the lowest and highest levels of income in drug use in primary health care. This is interesting since theses differences cannot be explained by differences depending on multimorbidity level between the different levels of income. Individuals with the lowest level of income had not the highest but next to highest odds ratio of having prescription drugs. This condition may be interpreted as if the individuals with the lowest level of income were unable to afford the prescribed drug, which has been seen before [6]. In Sweden, there is a high cost threshold system for prescription drugs, which implicates that the patients do not pay more than a defined amount for prescription drugs, SEK 2200 (EUR 248), annually. Despite this benefit system, it may be interpreted in this study as both individuals and patients with the lowest level of income did not purchase their prescribed drugs issued in primary health care.

The difference between the different income levels in odds ratio of having prescription drugs was larger when prescriptions issued by only GPs were examined compared to the differences seen in another of our studies where the prescription of drugs in the total population was examined [8]. This indicates that there is a social gradient in the way primary health care is provided.

### Education

Individuals and patients with the lowest level of education had the highest odds ratio and rate of prescription drug use issued in primary health care.

Former studies have shown that individuals with lower socioeconomic status appear to a lesser extent to act on information regarding health risks, e.g. smoking [32]. In many of our common chronic diseases, e.g. diabetes, hypertension and hyperlipidaemia, lifestyle changes are a first point of action, and if individuals with lower socioeconomic status do not act on the physicians’ recommendations of lifestyle changes, this may lead to both higher odds ratio and rate of drug use. Utilisation of health care differs according to socioeconomic status with a higher consultation rate among individuals with low socioeconomic status [33]. This may lead to increased odds ratio of having prescription drugs among individuals with lower socioeconomic status.

The difference between the different educational levels in odds ratio of having prescription drugs was also larger when prescription drugs issued by only GPs was examined compared to when the prescription of drugs in the total population was examined [8].

### Interactions

When data were analysed for each educational level the rate of prescription drug use among patients in every income level followed the same pattern as in Model 1. This means that also at different educational levels the income level still affects the rate of prescription drug use among patients.

Individuals with the lowest income level had the lowest odds ratio of having prescription drugs if they belonged to educational level 2 or above. This means that the poorest individuals use drugs to a lesser extent if they have completed only primary school. This elucidates further that there is a social gradient in the way primary health care is provided.

### Limitations

ACG Case-Mix uses diagnoses to calculate multimorbidity level. It is, therefore, dependent on the quality of registration of diagnoses. The recording of diagnoses was not validated in this study, but a former study in Sweden has shown that 75% of the population have at least one diagnosis-registered encounter with a GP during a three-year period [34].

Even if all the prescription drugs in this study were issued in primary health care, some of the prescriptions were probably originally initiated in secondary care. That is, some of the prescriptions are probably iterations from secondary care and hence do not necessarily entirely reflect the prescribing of drugs in primary care.

In this study we examined the prescription drugs that were collected from the pharmacies and not the prescription drugs that were actually prescribed by the doctors. If the compliance was inadequate, the collection of drugs does not adequately reflect the prescribing of drugs.

We were not able to assess illicit drug use, and these drugs were not included in this study.

It would have been interesting to include the level of general practitioners in the multi-level analysis but in the Swedish Prescribed Drugs Register we had access only to data down to Primary Health Care Centre level.

## Conclusion

There is a large difference in prescription drug use with regard to age, gender and socioeconomic status in primary health care after adjustment for multimorbidity. This implicates that the prescription drug use is not equal in society, and that factors other than medical ones affect the prescribing of drugs. This should be emphasised to both decision-makers and medical staff.

## Additional file

Additional file 1: Table S1. Odds ratios and incidence rate ratios of prescription drug use in different income levels by educational level after adjustment for gender, age and multimorbidity level. **Table S2.** Odds ratios and incidence rate ratios of prescription drug use in different multimorbidity levels (RUB 0-5) by age group after adjustment for gender and level of income.
