# Supplementary material for: Drugs prescribed by general practitioners according to age, gender and socioeconomic status after adjustment for multimorbidity level
Source: BMC Fam Pract. 2014 Nov 25;15:183. doi: 10.1186/s12875-014-0183-8 (PMC4246463; doi:10.1186/s12875-014-0183-8)
Supplement: Additional file 1: Table S1. — Odds ratios and incidence rate ratios of prescription drug use in different income levels by educational level after adjustment for gender, age and multimorbidity level. Table S2. Odds ratios and incidence rate ratios of prescription drug use in different multimorbidity levels (RUB 0-5) by age group after adjustment for gender and level of income. [file 12875_2014_183_MOESM1_ESM.docx]

Additional file 1: Table S1. Odds ratios and incidence rate ratios of prescription drug use in different income levels by educational level after adjustment for gender, age and multimorbidity level.

| Variables | OR (CI 95%) | p-value | IRR (CI 95%) | p-value |
| --- | --- | --- | --- | --- |
| Educational level 1 (low) |  |  |  |  |
| Income level 1 (low) | 1 |  | 1 |  |
| Income level 2 | 1.11 (1.03-1.20) | 0.011 | 0.98 (0.93-1.04) | 0.558 |
| Income level 3 | 0.94 (0.84-1.03) | 0.203 | 0.78 (0.72-0.85) | <0.001 |
| Income level 4 (high) | 0.86 (0.75-0.98) | 0.018 | 0.74 (0.68-0.80) | <0.001 |
|  |  |  |  |  |
| Educational level 2 |  |  |  |  |
| Income level 1 | 1 |  | 1 |  |
| Income level 2 | 1.21 (1.14-1.28) | <0.001 | 0.97 (0.86-1.08) | 0.543 |
| Income level 3 | 1.04 (0.98-1.10) | 0.161 | 0.69 (0.62-0.77) | <0.001 |
| Income level 4 | 0.98 (0.88-1.08) | 0.717 | 0.68 (0.63-0.74) | <0.001 |
|  |  |  |  |  |
| Educational level 3 |  |  |  |  |
| Income level 1 | 1 |  | 1 |  |
| Income level 2 | 1.16 (1.12-1.20) | <0.001 | 1.00 (0.94-1.06) | 0.929 |
| Income level 3 | 1.10 (1.05-1.15) | <0.001 | 0.81 (0.75-0.87) | <0.001 |
| Income level 4 | 1.06 (1.01-1.12) | 0.021 | 0.75 (0.70-0.80) | <0.001 |
|  |  |  |  |  |
| Educational level 4 (high) |  |  |  |  |
| Income level 1 | 1 |  | 1 |  |
| Income level 2 | 1.15 (1.07-1.22) | <0.001 | 1.12 (1.03-1.21) | 0.008 |
| Income level 3 | 1.19 (1.12-1.25) | <0.001 | 0.93 (0.87-0.99) | 0.032 |
| Income level 4 | 1.06 (1.01-1.13) | 0.079 | 0.85 (0.79-0.91) | <0.001 |

Additional file 1: Table S2. Odds ratios and incidence rate ratios of prescription drug use in different multimorbidity levels (RUB 0-5) by age group after adjustment for gender and

level of income.

| Variables | OR (CI 95%) | p-value | IRR (CI 95%) | p-value |
| --- | --- | --- | --- | --- |
| Age 20-39 |  |  |  |  |
| RUB 0 (low) | 1 |  | 1 |  |
| RUB 1 | 2.76 (2.67-2.85) | <0.001 | 0.61 (0.54-0.68) | <0.001 |
| RUB 2 | 3.25 (3.15-3.34) | <0.001 | 0.83 (0.75-0.92) | <0.001 |
| RUB 3 | 3.51 (3.41-3.62) | <0.001 | 1.21 (1.09-1.34) | <0.001 |
| RUB 4 | 4.30 (4.09-4.51) | <0.001 | 1.70 (1.40-2.06) | <0.001 |
| RUB 5 (high) | 4.32 (4.07-5.54) | <0.001 | 3.99 (1.88-8.45) | <0.001 |
|  |  |  |  |  |
| Age 40-59 |  |  |  |  |
| RUB 0 | 1 |  | 1 |  |
| RUB 1 | 2.40 (2.32-2.47) | <0.001 | 0.69 (0.64-0.75) | <0.001 |
| RUB 2 | 2.90 (2.83-2.96) | <0.001 | 0.91 (0.86-0.96) | 0.001 |
| RUB 3 | 3.45 (3.37-3.54) | <0.001 | 1.30 (1.24-1.37) | <0.001 |
| RUB 4 | 3.80 (3.67-3.93) | <0.001 | 1.77 (1.56-2.00) | <0.001 |
| RUB 5 | 3.82 (3.55-4.08) | <0.001 | 2.17 (1.86-2.54) | <0.001 |
|  |  |  |  |  |
| Age 60- |  |  |  |  |
| RUB 0 | 1 |  | 1 |  |
| RUB 1 | 2.26 (2.19-2.32) | <0.001 | 0.79 (0.74-0.85) | <0.001 |
| RUB 2 | 2.70 (2.59-2.81) | <0.001 | 0.98 (0.92-1.04) | 0.442 |
| RUB 3 | 3.22 (3.10-3.34) | <0.001 | 1.37 (1.30-1.44) | <0.001 |
| RUB 4 | 3.50 (3.33-3.68) | <0.001 | 1.82 (1.68-1.96) | <0.001 |
| RUB 5 | 3.71 (3.45-3.97) | <0.001 | 1.83 (1.59-2.10) | <0.001 |
